# Exploring Associations of Housing, Relocation, and Active and Healthy Aging in Sweden: Protocol for a Prospective Longitudinal Mixed Methods Study

**DOI:** 10.2196/31137

**Published:** 2021-09-21

**Authors:** Magnus Zingmark, Jonas Björk, Marianne Granbom, Giedre Gefenaite, Frida Nordeström, Steven M Schmidt, Taina Rantanen, Björn Slaug, Susanne Iwarsson

**Affiliations:** 1 Department of Health Sciences Lund University Lund Sweden; 2 Division of Occupational and Environmental Medicine Lund University Lund Sweden; 3 Faculty of Sport and Health Sciences Gerontology Research Centre University of Jyvaskyla Jyvaskyla Finland

**Keywords:** accessibility, activity, age-friendly housing, aging-in-place, housing preferences, life-space, mobility, moving expectations, neighborhood, participation

## Abstract

**Background:**

While housing and neighborhood features have the potential to impact opportunities for active aging, there is a lack of knowledge related to how older people reason regarding their housing situation and how housing and fulfillment of relocation are associated with active and healthy aging.

**Objective:**

The objectives of Prospective RELOC-AGE are to study housing choices and relocation and explore effects on active and healthy aging among men and women aged 55 years and older in Sweden considering relocation.

**Methods:**

The estimated sample (2800) will include people aged 55 years and older being listed for relocation at either of two housing companies: a local public housing company in Southern Sweden and a national condominium provider. Prospective RELOC-AGE has a 2-level longitudinal mixed methods design and includes quantitative surveys (implemented by a professional survey company) and a telephone interview for baseline data collection in 2021, with follow-ups with the same procedures in 2022 and 2023. The survey and interviews include questions related to present housing and neighborhood, relocation plans and expectations, a range of perspectives on active and healthy aging, and demographics. Linking to national registers will provide additional data on home help and health care use, objective housing, and neighborhood characteristics. To explore what housing attributes older adults considering relocation find important and to what extent when making their decisions on housing, we will develop a discrete choice experiment to be implemented with a subsample of participants. Further, a grounded theory approach will be applied to collect in-depth interview data from participants who have moved to another dwelling, within 6 months of the move. A follow-up interview 12 months later will focus on participants’ deepened experience over time in terms of fulfilled expectations and relocation experiences.

**Results:**

As of submission of this protocol (June 2021), recruitment has commenced with approximately 960 respondents to the survey and ongoing telephone interviews. We anticipate recruitment and data collection based on surveys and interviews to continue during 2021.

**Conclusions:**

Prospective RELOC-AGE has the capacity to generate new policy-relevant knowledge on associations of housing, relocation, and active and healthy aging. Such knowledge is relevant for the development of proactive approaches to housing in old age on the individual, group, and societal levels.

**Trial Registration:**

ClinicalTrials.gov NCT04765696; https://clinicaltrials.gov/ct2/show/NCT04765696

**International Registered Report Identifier (IRRID):**

DERR1-10.2196/31137

## Introduction

### Background

Previous research on housing and aging has mainly concerned frail older adults and their needs for residential care toward the end of life. According to the public debate, older people in general are interested in housing options that support active and healthy aging. However, comprehensive studies on housing options in later life incorporating health and social factors as well as factors related to the built environment and housing are lacking [1], and little is known about when and how people start to reflect and act upon housing choices and relocation as they age. Further, there is a lack of knowledge about how housing and relocation are related to active and healthy aging.

The body of recent literature on housing choices and relocation is limited, with interest for moves to special forms of housing at the core [2]. When comparing people remaining in ordinary housing with those moving to supported living in the format of retirement villages, those who did not move were initially better off, but after 3 months the difference decreased due to improvement among the movers, mostly in depression and self-rated health [3]. Somewhat in contrast, a British panel study showed that moving to residential housing was associated with higher mortality in the next 12 months among people aged 65 years and older, especially among men [4]. A study from Australia showed that reasons to move reflect the urge to maintain independence, stay in control, and avoid loneliness, and control over relocation decisions and being proactive contribute to positive adjustment [5].

There is ample evidence that housing is associated with health outcomes as people age, with some support for causal effects between housing and disability-related outcomes [6-9]. As an example, the association between housing accessibility and independence in activities of daily living seems to be mediated by external housing-related control beliefs in younger old [8]. Additional findings point to potentially different role of external housing-related control beliefs in different population groups, such as the very old people [7] or people aging with Parkinson disease [9], calling for further research in this area.

There are also qualitative studies showing that the home environment is important for activity and participation in very old age [10]. Noteworthy, perceived aspects of home are related to health already at age 67 to 70 years [11] with retirement stimulating active reflections regarding housing choices and relocation [12]. There are examples of quantitative cross-national studies of scale targeting neighborhoods and aging [13], but there is no population-level research with detailed data on objective and perceived aspects of housing as related to active and healthy aging.

Relocation has been described as a process negotiated over time [14] until turning points emerge [15]. Residential reasoning (eg, whether to move or not and how to arrange one’s housing situation) is a complex and ambivalent matter [16]. Changes in such reasoning relate to the way people strive to build upon or dismiss attachment to place and their attempts to maintain or regain residential normalcy during years of declining health [17]. Relating to such findings, different factors predict relocation to ordinary housing and residential care [17,18]. A study in the United States involving more than 7000 people aged 65 years and older [18] revealed that over a 4-year period, 8% moved within ordinary housing and 4% moved to residential care. Very old people who relocate do move to dwellings with fewer environmental barriers, but because of increasing functional limitations over time, housing accessibility problems persist [19]. Exemplifying complex dynamics of importance for housing choices and relocation in later life, very old people living in housing with more accessibility problems rate perceived meaning of home as worse and are more dependent on external control to manage their situation compared with younger older adults [20].

### Active Aging

Active aging is a policy goal referring to “the process of optimizing opportunities for health and participation in the society for all people in line with their needs, goals, and capacities as they age” [21]. Initiatives to promote active aging can be seen from a societal perspective in terms of providing accessible environments including transportation and housing or from a service provider perspective, for example, in terms of health-promoting interventions. In addition to the potential benefits on health, participation, and quality of life, the goal to promote active aging holds the potential to mitigate an expected increase in health and social care expenditures related to the increasingly larger proportion of older adults in the population.

Active aging can also be seen from an individual perspective in terms of strategies and behaviors that the individual can adopt to optimize their opportunities for participation and health. On the individual level, active aging has been described as striving for well-being through activity as per one’s goals, opportunities, and abilities [22]. One central, contextual facet of active aging is therefore housing and services that are tailored to address age-friendly housing and relocation and to support independent living [23]. However, in research on housing choices and relocation among older people, active aging has not been used as a core perspective or as an outcome to evaluate the long-term impact of housing and relocation. To inform the design of policies and societal support related to housing, knowledge is needed about how housing and relocation are associated with active aging and health outcomes.

### Study Objective and Research Questions

Nurtured by the hypothesis that housing choices and relocation influence opportunities for active and healthy aging, the objectives of Prospective RELOC-AGE are to study housing choices and relocation and explore effects on active and healthy aging among people aged 55 years and older in Sweden who are considering relocation. The specific research questions are:

- How do housing aspects and relocations affect future activity and health outcomes?
- What aspects of housing and health may explain or predict (1) relocation to different housing options in the ordinary housing stock, (2) relocation to residential care facilities, and (3) remaining in the present dwelling?
- What is the interaction between objective and perceived aspects of housing and social aspects associated with active and healthy aging, and what are the characteristics and trajectories of such dynamics?
- What housing attributes do older adults considering relocation find important and to what extent when making their decisions on housing preferences?
- How do older adults considering relocation decide regarding (1) different housing options and (2) motives for considering and effectuating relocation, and (3) to what extent are their motives fulfilled?
- How are the questions above affected by age, sex, civil status, country of origin, functioning, adverse health events, loss of a partner, and socioeconomic and neighborhood characteristics?

## Methods

The overall RELOC-AGE project comprises 3 parts: a population-based register study, a prospective mixed methods longitudinal study, and an intervention study. This paper is the study protocol for the prospective study.

### Study Design

**Figure 1:**
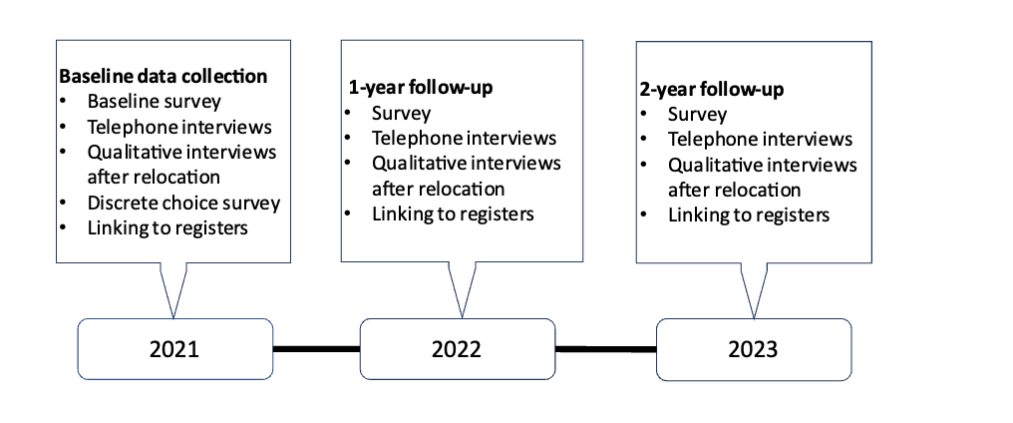
Overview of study design and time plan for data collection in Prospective RELOC-AGE.

Prospective RELOC-AGE has a 2-level longitudinal mixed methods design (Figure 1). Level 1 includes quantitative online surveys and a telephone interview for a baseline data collection in 2021 with follow-ups with the same procedures to be conducted in 2022 and 2023. To decrease participant burden, linking to registers will provide additional data. For level 2, we will retrieve relocation dates from collaborating housing companies or the Swedish Taxation Authority every third month to identify survey participants who have relocated to another dwelling (any type or form). They will be asked to participate in additional quantitative and qualitative data collection at home visits in their new dwelling or by telephone interview no later than 6 months after the move. User involvement is a significant component [24,25] engaging older adults and representatives from housing companies throughout the research process. The study was registered at ClinicalTrials.gov [NCT04765696] [26].

### Population and Setting

In order to capture dynamics related to housing choices, relocation, and active and healthy aging from an early stage of the aging process, age 55 years or older with a postal address in Sweden serve as inclusion criteria. Targeting people actively considering relocation, additional inclusion criteria are being voluntarily and actively listed based on interest for moving to a dwelling provided by either of 2 housing companies, selected based on established research collaboration. Severe cognitive impairments or insufficient language skills to give informed consent or participate in telephone interviews are exclusion criteria.

The housing companies represent a local public housing company (LPH) in Southern Sweden and a national condominium provider (NCP). In this way, a diversity of types of housing typically attracting people from different socioeconomic groups is represented. More specifically, the LPH provides common apartments as well as apartments designated for senior citizens. In February 2021, 1680 individuals aged 55 years and older were on the LPH waiting list. As condominiums are sold on the open market, NCP has priority and interest lists for new establishments of which some are designated for senior citizens. In February 2021, the NCP had approximately 22,000 individuals aged 55 years and older on their priority list and 33,000 on their interest list.

Based on the explorative overall objective and mixed methods design, the recruitment strives for inclusion of information-rich participants rather than representativity. The annual incidence of moves in the population aged 60 to 84 years is 4% to 5% [27]. Even if our sample will be younger, as we target a population actively considering relocation, we estimate a 50% higher relocation incidence. Thus, we will be able to study associations hitherto not addressed at scale in a population of older adults actively considering housing choices. Survey participants will fall into 5 categories to be compared with respect to health trajectories in the quasi-experimental design: still queuing for senior housing, moved to a regular dwelling in the ordinary housing stock, moved to needs-assessed residential care, received an offer in senior housing and moved, or deceased. The targeted sample size for the survey is 2800, which will be sufficient for the planned types of analyses. As an example, we have 90% statistical power (5% significance level) to detect if a certain lifestyle exposure, activity, or mobility pattern that is present among 20% of the participants increases the risk with 50% (risk ratio 1.5; 15% vs 10%) for multimorbidity during follow-up.

### Recruitment

Following the housing companies’ procedures to ensure that data were handled according to General Data Protection Regulations and based on written agreements between them and the research group, contact information for persons on their lists were either delivered to the university or provided directly by interested individuals via an online portal setup by the researchers. The recruitment process will be closely monitored and additional LPH companies will be approached to increase the recruitment base if necessary to reach the targeted sample size.

A stepwise recruitment procedure will include all eligible individuals from the LPH and randomly selected individuals from the NCP. A professional support organization for clinical and epidemiological research (Clinical Studies Sweden Forum South) with longstanding expertise on conducting surveys for research and handling data will implement the data collection.

An invitation letter will be sent by postal mail to the potential participants. The letter includes a description of the project, the methods for data collection, and how data will be handled and stored according to existing regulations. The information stresses that participation is voluntary and participants can decline participation at any time without consequences to their rights and access to be offered a dwelling offer or any societal services. The invitation letter includes information about alternative modes of answering the survey: a web-based survey to be accessed through the project website [28] with a participant-specific username and password provided in the invitation letter or a paper version of the survey to be sent to participants upon request.

### Ethics

Following the principles of the Helsinki Declaration and current national legislation and policies on ethics for research involving humans, Prospective RELOC-AGE was approved by the Swedish Ethical Review Authority (No. 2020-03457).

### Procedure

#### Survey Data Collection Procedures and User Involvement

The survey data collection includes a range of established instruments for studies on aging and housing and a study-specific discrete choice questionnaire aimed at exploring stated preferences on housing.

Nonacademic partner representatives and senior citizen representatives were engaged throughout the development of the data collection procedures. All data collection forms were piloted to optimize readability and the logic flow of questions and to minimize respondent burden. Such piloting was implemented in a stepwise manner and typically included 5 to 10 user representatives instructed to use different types of digital devices to respond to the online survey. Comments and suggestions for optimization were considered in the finalization of the data collection formats.

Most of the survey will be administered as a questionnaire, to be completed online or on paper. Due to the complex nature of the questions included, the University of Jyvaskyla Active Aging Scale, Meaning of Home, and External Housing-Related Control Beliefs Questionnaire will be administered during a telephone interview with participants who agree to this additional data collection.

##### Present Dwelling

Questions about the respondents’ present dwelling include type of dwelling (eg, apartment or house); whether the respondent owns their dwelling; if the entrance floor includes bathroom, bedroom, kitchen, place for dining, living room, hall, room for storage, and opportunities to wash and dry clothes (yes/no for each); number of rooms and rooms with opportunities to bath or shower; if there are stairs, ramp, or elevator at the entrance (yes/no); access to garden, balcony, or terrace (yes/no); if the dwelling is situated in an urban or rural area; the number of people living in the dwelling; cohabitants (eg, partner, children); year moved to present dwelling; and time per day spent out of the home.

##### Perceived Aspects of Housing and Neighborhood

Based on a model of perceived aspects of housing [29], the survey questionnaire includes 4 instruments with acceptable psychometric properties when applied in research on aging and housing.

Usability in the home is evaluated with selected items from the original Usability In My Home instrument [30,31]. The respondent rates to what extent they perceive the current dwelling is designed for managing personal activities of daily living (eg, bathing, toileting); food preparation; washing, cleaning, and flower care; and laundry and grooming (scale ranging from 1 to 5; higher = more usable).

Housing satisfaction [29] is evaluated with the question “Are you satisfied with your dwelling?” (scale ranging from 1 to 5; higher = more satisfied).

Meaning of home is evaluated with the Meaning of Home Questionnaire [29]. The concept is rooted in “What makes the house a home?” and focuses on the relationship between the sociophysical setting of the home and subjective evaluations, values, emotions, and goals. The questionnaire has a set of statements divided in 4 domains (physical, behavioral, cognitive/emotional, and social) rated on a scale from 0 to 10 how well they fit their own thinking (higher = more agreement). The instrument has adequate psychometric properties for use with adults aged 67 to 70 years in Sweden [32].

External Housing-Related Control Beliefs Questionnaire (HCQ) is evaluated with 16 items from the original HCQ questionnaire [33]. External control in relation to the home means that some other person, luck, chance, or fate are perceived as explanatory factors for what happens. For each item, respondents use a scale from 1 to 5 to rate to what extent they personally agree or disagree with the statements (higher = more external control). The external HCQ scale has adequate psychometric properties for use with adults aged 67 to 70 years in Sweden [32].

Neighborhood and outdoor experiences are evaluated with 2 sets of questions routinely used in regional public health surveys in Sweden [34]. The first set concerns access to societal services (eg, grocery shop, child care), cultural activities (eg, cinema, library), leisure facilities (eg, swimming hall), and public transportation and exposure to disturbing sounds and air pollution (yes/no/no opinion). The second set concerns 8 perceived qualities or characteristics of open green urban areas that can be described as serene, wild, lush, spacious, culture, the common, the pleasure garden/refuge, and festive [35,36] and one additional question about access to blue space (eg, lake, sea, water courses). Participants are asked to score each quality or characteristic within 5 to 10 minutes’ walking distance from their dwelling (4-point scale from totally disagree to totally agree). Five of the perceived items have been validated previously against objective landscape data [37].

Neighborhood cohesion is evaluated with a perceived neighborhood social cohesion scale from the National Health and Aging Trends Study [38] with 3 statements to which the respondent is asked to rate their level of agreement (not at all, to some extent, agree). The statements ask if people in their community know each other well, are willing to help each other, and can be trusted.

##### Relocation

A set of study-specific questions is used to capture reasons for considering relocation, moving expectations, and previous moving experiences. What were the reasons to apply for being on a waiting/interest list for another dwelling (several response alternatives, eg, I do not want or am unable to manage my present dwelling; I want a dwelling that provides better opportunities for me to engage in activities I prefer to do)? When is it likely that you will move (in less than 1 year, 1 to 2 years, 2 years or more)? How likely is it that you have moved within 2 years (on a 5-point scale from 1, completely certain, to 5, not at all likely)? What kind of dwelling would you like to move to (eg, rented or owned apartment, house)? To what extent will a decision to relocate be made by the respondent themself, together with or by others? Are there hindrances to relocate within 2 years (eg, economic reasons, poor health)? How many times have you moved since the age of 18 years? Can housing adaptations be an option rather than moving?

##### Active and Healthy Aging

Self-rated health is evaluated with the widely used 1-item question from the SF-12 scale [39], “In general, would you say your health is...” (5 response options ranging from poor to excellent).

Illness, disease, and recent health care use is evaluated with study-specific questions on whether the respondent has any type of long-term illness or disease (yes/no), and if so, if that has an impact on work or daily activities (yes/no); if the respondent has ever been diagnosed with depression by a medical doctor (yes, during the previous 12 months, yes, more than 12 months ago, no); whether the person has been admitted to hospital (yes/no) or visited an emergency department (yes/no) during the past 3 months.

Functional limitations are evaluated using 10 items on functional limitations (rated as present, yes, to some extent, or not present) adapted from the person component of the Housing Enabler instrument [40].

Mobility questions related to opportunities for mobility were developed after consultation with a researcher specializing in mobility issues involving older adults. Study-specific questions included whether the respondent has a driver’s license (yes/no), access to a car (yes/no), the potential and realized use of (can you... and do you ... respectively): walk, bike, moped/motorcycle, car, train, bus, transportation service, subway/tram, or ferry [41]. Satisfaction with mobility opportunities is rated on a 5-point scale (from very satisfied to very dissatisfied).

Physical activity is evaluated with a question from a well-established public health survey [34] about the total time per week the respondent is physically active (eg, brisk walking, gardening; 6 levels ranging from 0 minutes to 5 hours or more per week). Physical exercise [34] is evaluated with a question from the same survey about the total time per week the respondent is engaged with strenuous activities (5 levels ranging from 0 minutes to 2 hours or more per week).

Use of technical aids is evaluated with 5 study-specific questions about the use of a cane, crutches or similar, rollator, manual wheelchair, electric wheelchair, or scooter (no, yes outdoors, yes indoors).

Life-space mobility is evaluated with the Swedish version [42] of the Life-Space Assessment [43], which includes 5 levels of life-space mobility and whether the respondent, during the previous 4 weeks, has been to any of these locations: indoors to other rooms than the bedroom, immediate outdoor surroundings, neighborhood, town, or beyond town. For each level, the respondent indicates how often (less than once per week, 1 to 3 times per week, 4 to 6 times per week, every day), and if they needed a technical aid or assistance. The composite score ranges from 0 to 120; higher scores indicate greater life-space mobility.

Active aging is evaluated with the University of Jyvaskyla Active Aging Scale [22,44]. This instrument contains 17 self-rated items regarding goals, ability, autonomy, and activity that capture a single construct reflecting individual active aging [22] (total score ranges from 0 to 272). The items include practicing memory, using a computer, advancing matters in one’s own life, exercising, enjoying the outdoors, taking care of one’s personal appearance, crafting or DIY, making one’s home cozy and pleasant, helping others, maintaining friendships, getting to know new people, balancing personal finances, making one’s days interesting, practicing artistic hobbies, participating in events, advancing societal/communal matters, and doing things in accord with one’s world view [45].

Self-rated health is evaluated with the EQ-5D-5L [46], which includes the items mobility, washing and dressing, and daily activities, which are rated on a 5-point scale with higher scores indicating a worse health status. If the respondent rates at least moderate difficulty on one or more of these 3 items, the respondent is also presented questions about frailty below. Further, the EQ-5D-5L includes items regarding if the respondent experiences pain/discomfort or anxiety/depression; both are rated on a 5-point scale with higher scores indicating a worse health status.

Frailty is evaluated by 4 questions (yes/no) [47]: Have you had any general fatigue or tiredness over the last 3 months? Do you fall often, or are you afraid of falling? Do you need assistance in either getting to the store, managing obstacles to and from the store, or in choosing, paying for, or bringing home groceries? Do you get tired when taking a 15- to 20-minute walk outside?

Life satisfaction is evaluated with the 1-item question, “How satisfied are you with life as a whole?” (6 response options, from very unsatisfying to very satisfying) [48].

Self-efficacy is evaluated with the general self-efficacy scale [49], which includes 10 statements (eg, I always manage to solve problems if I make an effort to do it; In unexpected situations I always know how to act). For each statement, respondents state their agreement on a 4-point scale from 1, completely disagree, to 4, totally agree.

Receiving or providing practical support in daily life is evaluated with a set of study-specific yes/no questions: Do you receive practical support in your daily life from a family member? Do you in your daily life provide practical support for a family member with health or functional limitations in their daily life? Do you have a safety alarm? Do you receive home help? Do you live together with someone who receives home help? Have you received practical support in or outside your house during the last 2 months? If yes, was the support from a family member, neighbor, or friend; municipality handyman; home help; or a private company?

Life events are evaluated with study-specific questions about experiencing major life events during the previous 3 years (yes/no): death of a spouse/partner, own disease, disease/disability of a spouse/partner, disease/disability of other close person, divorce/separation, became grandparent, got married/registered partnership, reduced time working/or retiring, begun to work, or driving cessation.

##### Demographics

Demographic questions include civil status, Swedish origin (if not, age when coming to Sweden), gender, educational, current occupation, and economic situation.

##### Stated Preferences on Housing

To explore stated preferences and the importance of various housing attributes when considering relocation, we will conduct a discrete choice experiment (DCE) [50]. A DCE is a quantitative technique for eliciting individual, stated preferences, in this study in relation to housing. Stated preferences have been used to examine housing decisions [51] among tenants in general [52] but not in aging research. A key feature of a DCE is to identify attributes based on existing literature and expert or user consultations. We will develop the DCE using an iterative process including a review of literature, expert consultations, and user involvement. Potential attributes include location, accessibility, costs, distance to bus stops, and services in the local neighborhood [53]. In a DCE, respondents are presented different hypothetical alternatives where the degree to which important attributes are present varies, followed by responses regarding how different alternatives are valued in relation to each other. The DCE included in Prospective RELOC-AGE is under development and will be presented in a forthcoming publication.

Table 1 provides an overview of instruments and study-specific questions.

**Table 1:** Overview of instruments and study-specific questions used in the survey study.

Question/instrument		Source	Baseline	1 year	2 years
**Housing and relocation**					
	Present dwelling	Study-specific	✓	✓	✓
	Usability in the home	[30,31]	✓	✓	✓
	Housing satisfaction	[29]	✓	✓	✓
	Meaning of home	[29,32]	✓	✓	✓
	Housing-related control beliefs	[32,33]	✓	✓	✓
	Neighborhood cohesion	[38]	✓	✓	✓
	Neighborhood and outdoor experiences	[35,36]	✓	✓	✓
	Reasoning around relocation	Study-specific	✓	✓	✓
	Moving expectations	Study-specific	✓	✓	✓
	Relocation experiences	Study-specific	✓	✓	✓
**Active and healthy aging**					
	Self-rated health	[39]	✓	✓	✓
	Illness, disease, recent health care use	Study-specific	✓	✓	✓
	Functional limitations	[40]	✓	✓	✓
	Physical exercise	[34]	✓	✓	✓
	Physical activity	Selected questions from public health survey [34]	✓	✓	✓
	Use of technical aids	Study-specific	✓	✓	✓
	Life-space mobility	[42,43]	✓	✓	✓
	Active aging	[22]	✓	✓	✓
	Self-rated health	[46]	✓	✓	✓
	Frailty	[47]	✓	✓	✓
	Life satisfaction	[48]	✓	✓	✓
	Self-efficacy	[49]	✓	✓	✓
	Caregiving	Study-specific	✓	✓	✓
	Mobility	Study-specific modified from [41]	✓	✓	✓
	Life events	Study-specific	✓	✓	✓
**Demographics**					
	Current occupation	Study-specific	✓	✓	✓
	Economic situation	[34]	✓	✓	✓
**Stated preferences on housing**					
	Discrete choice experiment	Study-specific	✓	—^a^	—

^a^Not applicable.

#### Postrelocation In-Depth or Semistructured Interviews

Over time, survey participants who have moved to another dwelling (any type/form) will be asked to participate in an in-depth interview no later than 6 months after the move. Using a grounded theory approach [54,55], we will develop an interview guide focusing on the relocation experience. Performing data collection and analysis in parallel to determine the need for additional sampling, based on the principle of saturation [56] the sample size is not predetermined. Trained research staff will collect data at home visits or online, depending on what is feasible at the time for the data collection. Approximately 12 months after the first in-depth interview, a subsample of typical cases (estimated at 25) will be selected based on the initial in-depth interview. The follow-up interview will deepen the knowledge about the relocation experience fulfillment of expectations over time.

#### Complementary Data by Linking to National Registers

In order to decrease participant burden and bias related to self-reporting, we will use complementary health and housing data for each time point in the data collection requested for the Register RELOC-AGE Study. These data are made available through Statistics Sweden (eg, the Total Population Register), the National Board of Health and Welfare (eg, National Patient Register), the Municipal Health Care Register, the Real Estate Property Register, and the Apartment Register. Data accessed from registers will concern objective housing data (eg, dwelling unit size), individual- and neighborhood-level demographic and socioeconomic indicators, health care and home help service use as well as causes of death.

### Data Analysis Plan

For quantitative data collected by surveys and phone interviews, we will apply exploratory and inferential statistical methods. For longitudinal analyses, we will use regression techniques including generalized linear models or Cox regression with time-dependent covariates. We will investigate how different personal and neighborhood-level characteristics affect the associations of interest by exploring mediation and moderation effects, as well as use different techniques to address confounding.

For analyses of data from the discrete choice experiment, we will use the conditional multinomial logit model as the reference model, but the analysis will be extended to mixed logit and latent class models to take into account preference heterogeneity [57]. The 2 latter models take into account the panel structure of the data and are a standard extension of the analysis [58,59].

In-depth interviews will be audiorecorded and transcribed, followed by analyses guided by principles from grounded theory [55] aided by the NVivo (QSR International) software.

## Results

As of submission of this protocol (June 2021), recruitment has commenced with approximately 960 respondents to the survey and telephone interviews ongoing. We anticipate recruitment and data collection based on surveys and interviews to continue during 2021.

## Discussion

### Summary

Prospective RELOC-AGE will provide new knowledge about whether and how housing choices and relocation have an impact on active and healthy aging among people aged 55 years and older in Sweden who are considering relocation. Following a large sample of information-rich individuals over time including a 2-level data collection in a mixed methods design, the results will add knowledge about associations between housing choices, perceived and objective aspects of housing and neighborhood, a range of socioeconomic factors, health, and active aging. Further, based on the explorative mixed methods approach, the project will contribute to a better understanding of factors that may explain or predict relocation or remaining in the present dwelling.

In housing-related aging research, the concept of aging-in-place is prominent but insufficiently problematized and currently geared toward health care, social services, and residential care needs [60]. The underlying premises are that the vast majority of older adults prefer to age in place [61], and it is less costly to provide care at home than in institutions [62]. Current research has a strong focus on people aging into disability and frailty with increasing needs for special forms of housing, tailored home modifications, or other reactive solutions at the core. The prevailing definition does not relate to proactive public health ambitions and strategies to support active and healthy aging. Results of cross-national research on aging and housing show that aging-in-place is far from applicable to all senior citizens [63]. In the light of such results as well as policies emphasizing the diverse needs of the heterogeneous aging population [64], the static and generalized notion of aging-in-place is facing a dead end. Integrating active and healthy aging with housing and relocation, RELOC-AGE challenges aging-in-place and the prevailing paradigm in this research field and will produce new knowledge for research as well as practice and policy.

As to the association between housing and health, previous research has mainly been focused on very old people (eg, qualitative studies showing that the home environment is important for activity and participation in very old age) [10]. However, as shown by Kylén et al [11], perceived aspects of home are related to health already at ages 67 to 70 years. In line with previous findings showing that processes close to the retirement age seem to stimulate active reflections regarding housing choices and relocation [12], the RELOC-AGE project is designed to capture such processes including a relatively young cohort that will be followed over time. An essential aspect of active aging is the opportunity and ability to engage in prioritized activities [22]. As such, housing and the neighborhood provide a starting point for engagement in such activities. At retirement age, people plan for self-realization, and thus have housing preferences different from those at more advanced ages, where compromised functional capacity and frailty may influence where people wish to live.

In Prospective RELOC-AGE, housing is not limited to the dwelling itself but refers to the location of the dwelling as well, thus including neighborhood features. The research field of natural outdoor environments and health has grown in the past decades [65], contributing to a better understanding about the existing links. In a cross-sectional study, serenity, wilderness, species richness, spaciousness, and cultural history were associated with neighborhood satisfaction, physical activity, and general health [66,67]. Moreover, perceived safety was shown to be a prerequisite for the association between the outdoor qualities and physical activity [68], confirming that the pathways between features in the outdoor environment and health outcomes are complex. While barriers to outdoor mobility located close to the home have been found to be associated with lower physical activity among older adults, barriers further away from home were not [69]. In addition, attractive destinations for outdoor mobility located at least 500 meters away from home were correlated with higher physical activity. While features in the neighborhood as well as in the dwelling provide opportunities or hindrances for engagement in activities that relate to a person’s goals, the association to active aging remains to be explored. Complementing existing research in this field, Prospective RELOC-AGE will shed new light on whether and how housing and relocation impact on active and healthy aging.

The longitudinal approach of Prospective RELOC-AGE in combination with Register RELOC-AGE will provide data that can be used to build causal evidence when it comes to housing and health associations. Self-reported and registry-based data on housing, demographics, and individual facets of active and healthy aging will enable us to explore potential mechanisms of how housing could support active and healthy aging. Such knowledge is essential to develop evidence-based future housing practices and policies in Sweden as well as abroad.

Previous intervention research related to housing issues typically has targeted home modifications, indicating that individual strategies promote participation among people with health conditions [70]. However, to the best of our knowledge, evidence-based interventions with a health promotion approach targeting housing matters before people are frail and need residential care do not exist. Parallel to Prospective RELOC-AGE and drawing on the knowledge gained, a web-based housing counseling intervention will be finalized and piloted (Intervention RELOC-AGE). The Aging in the Right Place was developed by using research circles [24] involving senior citizens, technology experts, and nonacademic partners. The existing prototype includes 3 modules: THINK, LEARN, and ACT, reflecting different stages of the decision-making process related to housing choices and relocation [71]. The knowledge gained from Prospective RELOC-AGE will contribute to further development and the finalization of the Aging in the Right Place intervention, which will subsequently be piloted and evaluated in municipality contexts in Sweden.

### Limitations and Strengths

Currently, Prospective RELOC-AGE is limited to a follow-up period of 2 years, which, given the complex associations between housing and relocation and active and healthy aging, could be considered too short. However, the planned follow-up period at this stage is determined based on available funding and will be extended as soon as additional funding has been secured. Thus, the ambition is to establish a solid structure for long-term follow-up, which is required to produce valid results responding to the ambitious research questions.

The 2-level mixed methods design could be seen as a strength as well as a limitation [72]. For example, given the exploratory design, the survey sample will not be representative for the age 55 years and older population in Sweden. The main reason for this is that we want to recruit a sample of information-rich individuals who are actively considering relocation. In Sweden, relocation rates in old age are in general quite low, which implies that in a representative sample of people as young as 55 years, very few could be considering relocation and even fewer actually realizing a move during the period of study. One way to ascertain that the sample includes diversity in terms of socioeconomic characteristics is to recruit participants via an LPH company as well as an NCP. Moreover, the sample will be geographically dispersed across the entire country. Being the first study of its kind, nationally as well as internationally, we consider the 2-level mixed methods design and sampling strategy promising and appropriate to expand the knowledge base on housing, relocation, and active and healthy aging even though results will not be generalizable to the whole population of older people [73]. However, we still anticipate that the longitudinal approach of Prospective RELOC-AGE has potential to yield results regarding housing and health associations that have high internal validity, but it is important that the risk of selection bias jeopardizing validity is assessed for each investigated association separately [74].

The data collection for Prospective RELOC-AGE is being implemented during a period when the COVID-19 pandemic is still affecting people of all ages as well as the society overall. How and to what extent the current situation will influence the data collected will be considered in the analyses and interpretation of results, as well as in the planning of subsequent follow-ups.

### Conclusion

Building upon well-established cooperation with nonacademic partners, this large and complex project has the capacity to generate new knowledge and policy-relevant results. The 2-level mixed methods design is novel and challenging, using a combination of quantitative and qualitative data collection methods that will generate data on hitherto understudied associations between housing and active and healthy aging. Such knowledge is relevant for the development of proactive approaches to housing in old age on the individual, group, and societal levels.

## Appendix

Multimedia Appendix 1 Peer-reviewer report from the Swedish Research Council (Vetenskapsrådet).

Multimedia Appendix 2 Peer-reviewer report from Forte: the Swedish Research Council for Health, Working Life and Welfare.

## Abbreviations

DCE: discrete choice experiment
HCQ: Housing-Related Control Beliefs Questionnaire
LPH: public housing company in Southern Sweden
NCP: national condominium provider
