# Supplementary material for: Exploring Associations of Housing, Relocation, and Active and Healthy Aging in Sweden: Protocol for a Prospective Longitudinal Mixed Methods Study
Source: JMIR Res Protoc. 2021 Sep 21;10(9):e31137. doi: 10.2196/31137 (PMC8493467; doi:10.2196/31137)
Supplement: Multimedia Appendix 1 [file resprot_v10i9e31137_app1.pdf]

Vetenskapsrådet

Beredningsgruppens yttrande

2019-00996	Susanne Iwarsson	Beredningsgrupp: MH-H1

Utlysningsnamn: Forskningsbidrag Stora utlysningen 2019 (Medicin och hälsa)

Projekttitel (svenska): RELOC-AGE: Vilken betydelse har val av boende och flyttningar för aktivt och hälsosamt åldrande?

Bidragsform: Projektbidrag

Sökt inriktning: Fri

Scientific quality of the proposed research 5

1 - Poor, 2 - Weak, 3 - Good, 4 - Very Good, 5 - Very good to excellent, 6 - Excellent, 7 - Outstanding

This is an important project. A strength is the use of several different methods. The methods are presented adequately. The project is limited to only three municipalities in the south of Sweden, which limits the representativity.

Novelty and originality 5

1 - Poor, 2 - Weak, 3 - Good, 4 - Very Good, 5 - Very good to excellent, 6 - Excellent, 7 - Outstanding

There is a great need of new knowledge in the field. This project will add new and important knowledge and will contribute to new and better methods to further improve research within this complicated research-field.

Merits of the applicant 5

1 - Poor, 2 - Weak, 3 - Good, 4 - Very Good, 5 - Very good to excellent, 6 - Excellent, 7 - Outstanding

Very good to excellent merits of the PI and a competent research-group with different competences important for the methods planned to be used.

Feasibility 3

1 - Not feasible, 2 - Partly feasible, 3 - Feasible

With perspective of previous experience in the research-group they will be able to perform this large and complex project.

Overall assessment of the application's scientific quality* 5

1 - Poor, 2 - Weak, 3 - Good, 4 - Very good, 5 - Very good to excellent, 6 - Excellent, 7 - Outstanding

New important project of high scientific quality which will add new knowledge to a research area with great needs of new knowledge.
