# Supplementary material for: Exploring Associations of Housing, Relocation, and Active and Healthy Aging in Sweden: Protocol for a Prospective Longitudinal Mixed Methods Study
Source: JMIR Res Protoc. 2021 Sep 21;10(9):e31137. doi: 10.2196/31137 (PMC8493467; doi:10.2196/31137)
Supplement: Multimedia Appendix 2 [file resprot_v10i9e31137_app2.pdf]

FORTE: Forskningsrådet för hälsa, arbetsliv och välfärd

Beredningsgruppens yttrande

**2019-01104**	**Susanne Iwarsson**	**Beredningsgrupp: Äldre19**

**Utlysningsnamn:** Forskning om åldrande och hälsa 2019	**Bidragsform:** Projekt
**Projekttitel (svenska):** RELOC-AGE: Vilken betydelse har val av boende och flyttningar för aktivt åldrande?	**Sökt inriktning:** Äldre

Bedömning

Syfte, frågeställningar, teorianknytning, bakgrund och projektets originalitet

This project aims at understanding the relationship between housing preferences, active ageing and wellbeing along persons aged 55 and over in Sweden. The background literature is discussed well, as are the relevant theories to this area of study. The project is related to an existing research project funded by the Swedish Research Council. The panel were impressed by the potential links between this project and other academic and non-academic partners.

Studiedesign, metoder för datainsamling och analys

The project aims to use mixed methods, focusing on the collection of primary data for quantitative analysis, and conducting qualitative interviews in groups with older people in order to elicit more information about their housing preferences. The methodology is clearly geared towards addressing the research questions.

Genus- och mångfaldsperspektiv i forskningens innehåll

The gender and diversity aspects have been considered carefully by the team and discussed well in the proposal.

Genomförbarhet

The project is broken down into carefully thought work packages, with particular members of staff allocated to each. The team are well placed to complete this project successfully, having worked in similar areas in the past.

Relevans, samverkan och nyttiggörande

The research team bring strong links with non-academic partners, such as a number of municipalities involved in the research. The project includes an innovative idea of a World Cafe which brings together users and researchers to discuss the project key findings. Including a PhD with four papers is an excellent idea and adds to the capacity building of the project.

Sammanfattande bedömning

Overall this is an interesting and policy-relevant project, reflecting a strong team and a plan which is realistic and do-able.

Förslag till beslut (bevilja, bevilja i mån av medel, avslå/approve, approve subject to funding, reject)

Approve
